# Adenoviral-Mediated Glial Cell Line–Derived Neurotrophic Factor Gene Transfer Has a Protective Effect on Sciatic Nerve Following Constriction-Induced Spinal Cord Injury

**DOI:** 10.1371/journal.pone.0092264

**Published:** 2014-03-18

**Authors:** An-Kuo Chou, Ming-Chang Yang, Hung-Pei Tsai, Chee-Yin Chai, Ming-Hong Tai, Aij-Li Kwan, Yi-Ren Hong

**Affiliations:** 1 Department of Anesthesiology, Kaohsiung Chang Gung Memorial Hospital and Chang Gung University College of Medicine, Kaohsiung, Taiwan, R.O.C.; 2 Department of Biological Sciences, National Sun Yat-Sen University, Kaohsiung, Taiwan, R.O.C.; 3 Department of Biochemistry, Faculty of Medicine, College of Medicine, Kaohsiung Medical University, Kaohsiung, Taiwan, R.O.C.; 4 Laboratory of Medical Research, Kaohsiung Armed Forces General Hospital, Kaohsiung, Taiwan, R.O.C.; 5 Graduate Institute of Medicine, College of Medicine, Kaohsiung Medical University, Kaohsiung, Taiwan, R.O.C.; 6 Department of Pathology, Kaohsiung Medical University Chung-Ho Memorial Hospital, Kaohsiung, Taiwan, R.O.C.; 7 Institute of Biomedical Science, National Sun Yat-sen University, Kaohsiung, Taiwan, R.O.C.; 8 Department of Neurosurgery, Kaohsiung Medical University Chung-Ho Memorial Hospital, Kaohsiung, Taiwan, R.O.C.; Universidade do Estado do Rio de Janeiro, Brazil

## Abstract

Neuropathic pain due to peripheral nerve injury may be associated with abnormal central nerve activity. Glial cell-line-derived neurotrophic factor (GDNF) can help attenuate neuropathic pain in different animal models of nerve injury. However, whether GDNF can ameliorate neuropathic pain in the spinal cord dorsal horn (SCDH) in constriction-induced peripheral nerve injury remains unknown. We investigated the therapeutic effects of adenoviral-mediated GDNF on neuropathic pain behaviors, microglial activation, pro-inflammatory cytokine expression and programmed cell death in a chronic constriction injury (CCI) nerve injury animal model. In this study, neuropathic pain was produced by CCI on the ipsilateral SCDH. Mechanical allodynia was examined with von Frey filaments and thermal sensitivity was tested using a plantar test apparatus post-operatively. Target proteins GDNF-1, GDNFRa-1, MMP2, MMP9, p38, phospho-p38, ED1, IL6, IL1β, AIF, caspase-9, cleaved caspase-9, caspase-3, cleaved caspase-3, PARP, cleaved PARP, SPECTRIN, cleaved SPECTRIN, Beclin-1, PKCσ, PKCγ, iNOS, eNOS and nNOS were detected. Microglial activity was measured by observing changes in immunoreactivity with OX-42. NeuN and TUNEL staining were used to reveal whether apoptosis was attenuated by GDNF. Results showed that administrating GDNF began to attenuate both allodynia and thermal hyperalgesia at day 7. CCI-rats were found to have lower GDNF and GDNFRa-1 expression compared to controls, and GDNF re-activated their expression. Also, GDNF significantly down-regulated CCI-induced protein expression except for MMP2, eNOS and nNOS, indicating that the protective action of GDNF might be associated with anti-inflammation and prohibition of microglia activation. Immunocytochemistry staining showed that GDNF reduced CCI-induced neuronal apoptosis. In sum, GDNF enhanced the neurotrophic effect by inhibiting microglia activation and cytokine production via p38 and PKC signaling. GDNF could be a good therapeutic tool to attenuate programmed cell death, including apoptosis and autophagy, consequent to CCI-induced peripheral nerve injury.

## Introduction

Neuropathic pain is caused by lesions or diseases of the somatosensory system including peripheral nerve injury and central nerve injury. Spontaneous pain, thermal-mediated hyperalgesia and tactile-evoked allodynia are common neuropathic pain symptoms following peripheral nerve injury, and significantly reduce quality of life and functional status. In clinical observation, neuropathic pain is not confined to the innervation area of the injured nerve, but also affects the adjacent area innervated by other intact nerves. Previous data have shown that sprouting from lamina III into II in neuronal remodeling in the spinal cord might result in the development of persistent tactile allodynia [1], [2]. Recent studies have demonstrated that C-fibers appear not to sprout outside their normal laminar distribution after injury [3]. According to current clinical experience, patients with neuropathic pain and visceral pain commonly have poor response to ordinary medication, and usually depend on opioid drugs for pain control [4]. Unfortunately, long-term administration of opiates has well-known side effects including drug addiction and tolerance, immunosuppression, and decreased micturition reflex. New therapeutic approaches such as gene therapy with pain-killer genes may hold promise for treating such patients.

Glial cell line-derived neurotrophic factor (GDNF) is one of the GDNF family of ligands (GFLs). GFLs are important for cell survival, neurite outgrowth, cell differentiation and cell migration, and GDNF promotes the survival of dopaminergic neurons [5]. Nerve injury downregulated GDNF and its receptor, GDNF family receptor alpha-1 (GDNFRa-1), on dorsal root ganglia [6]. Continuous injection of GDNF by osmotic pump promotes regeneration of sensory axons and attenuates neuropathic pain in animal models of nerve injury [7]–[9]. GDNF has been used as a therapy for neurodegenerative diseases such as Parkinson's disease [10], [11] and amyotrophic lateral sclerosis [12], [13]. However, the underlying molecular mechanism by which GDNF ameliorates neuropathic pain remains largely unknown. A better understanding of microglial-neuronal interactions in the SCDH will further our understanding of neural plasticity and may also lead to novel therapeutics for chronic pain management.

In this study, we used CCI as neuropathic pain model with adenoviral-mediated GDNF to evaluate the therapeutic effect of GDNF on peripheral nerve injury-induced neuropathic pain, analyzing protein expressions and activations in different aspects including microglia activation (MMP2, MMP9, p38, phospho-p38, IL6 and IL1β), caspase-dependent apoptotic markers (caspase-9, cleaved caspase-9, caspase-3, cleaved caspase-3, PARP, cleaved PARP), caspase-independent apoptotic markers (AIF, SPECTRIN and cleaved SPECTRIN), autophagy marker (Beclin-1), and CCI-induced proinflammatory markers (PKCσ, PKCγ, iNOS, eNOS and nNOS) to determine whether adenoviral-mediated GDNF gene therapy can successfully ameliorate the above gene expression and the different types of associated programmed cell death.

## Materials and Methods

### Animal model

Male Sprague–Dawley rats weighing (140 to 160 g at the time of surgery (NSC Animal Center, Taiwan) were fed with standard lab rodent chow and water *ad libitum* and housed individually. Rats were anesthetized with an intraperitoneal (i.p.) injection of sodium pentobarbital (Nembutal, 50 mg/kg), and CCI to the right sciatic nerve (SN) was done according to the method of Bennett and Xie (1988) [14], in which the left common sciatic nerves were exposed in the left midthigh and loosely ligated with 4-0 silk thread in three regions at about 1-mm intervals. Animal procedures were performed according to a protocol approved by the Institutional Review Board and Institutional Animal Care and Use Committee of Chang Guang Memorial Hospital.

### Gene therapy

Recombinant adenovirus vectors encoding GDNF (Ad-GDNF) or enhanced green fluorescent protein (Ad-GFP) were prepared as described previously [15]. For Ad-GDNF, GDNF cDNA was subcloned into pCA13 to yield the transfer vector, Ad5-GDNF, which was used to transfect 293 cells with pJM17, a plasmid containing the entire adenoviral genome, to generate recombinant virus through homologous recombination by calcium phosphate protocol as described previously [16]. The virus was amplified in 293 cells, purified by two rounds of cesium chloride gradient ultracentrifugation, and dialyzed against buffer containing 10 mM Tris, pH 7.5, 1 mM MgCl_2_, and 10% glycerol at 48°C. The titer of the virus solution was determined by measuring optical density at a wavelength of 260 nm and plaque-forming assay in 293 cells before storage at −80 °C. Adenovirus vectors [2×10^9^ plaque-forming units (pfu) in 100 ml sterile phosphate buffer saline PBS] were administrated via the triceps brachii muscle of anesthetized rats using a disposable insulin syringe equipped with a 27-gauge needle. Injection was performed in a biosafety P2 laboratory, and the care of animals receiving the adenovirus vectors conformed to institutional guidelines.

### Tissue preparation

The sciatic nerves were dissected and harvested at 7, 14, or 28 days after the CCI operation and at 28 days after viral injection paired with the CCI operation (six animals for each time point and group). The injured side of the sciatic nerve was cut into two segments from the region of ligation, the adjacent proximal and distal segments. The uninjured contralateral sciatic nerve in each animal was used as the control.

### Thermal hyperalgesia

Thermal sensitivity of the plantar hind paws was tested according to Hargreaves' method [17] using a Plantar Test Apparatus (Ugo Basile, Comerio, Italy). Rats were placed unrestrained in individual clear plastic compartments (11 cm×17 cm×14 cm). When the rats were stationary and not attending to the tester or stimulus, an infrared radiant heat source (180 mW) was applied through a glass floor to the middle of the plantar surface of the hind paw, between the foot pads. A photocell automatically stopped the heat source and the timer when the rat lifted its paw. For transected mice that were not capable of hind paw plantar placement, the rats were held gently to assist the plantar placement of the hind paws. Each rat was tested for five trials on each hind paw, with at least 1 min between trials, and the order of testing was randomized to minimize windup or avoidance behaviors. A 20-s maximum cutoff was established to prevent tissue damage.

### Allodynia test

The hind paw withdrawal threshold to tactile stimulation was determined using factory-calibrated Touch Test filaments (von Frey, Semmes–Weinstein monofilaments) (Stoelting, Wood Dale, IL, USA). Mice were placed under a small, clear compartment (8 cm×12 cm×5.5 cm) on an elevated wire mesh screen to allow the investigator free access to the plantar surface of the paws. For transected mice that were not capable of hind paw plantar placement, the mice were held gently to assist the plantar placement of the hind paws. Left and right hind paws were tested in a random order using the up-down method when the rat was not attending to the tester or the stimulus.

### Hematoxylin/eosin staining

Slides were counterstained with hematoxylin and eosin (H&E) as described elsewhere [18] for tissue examination. Briefly, 6 μm sections were deparaffinized in Xylol (Carl-Roth, Germany) for 10 minutes, rehydrated in a descending ethanol series and rinsed in deionized H_2_O for 1 minute. Sections were placed in hematoxylin for 3 minutes, rinsed in tap water for 1 minute to allow stain to develop and then placed in eosin for 1 minute, dehydrated and mounted in Entellan resin (Merck, Germany). The occurrence of clearly detectable eosinophilic spheroids indicative of dystrophic axons [19] was quantified in approximately 90 sections from ipsilateral SCDH so irregular results due to random deviations in spheroid numbers could be ruled out. H&E stained axonal spheroids were generally eosinophilic and round or oval in shape. They varied in diameter (5–50 μm) and sometimes reached a size larger than the nerve cells in SCDH. Morphology and density of neurons within the spinal cord were assessed. To avoid examining the same neurons twice, we left more than an 8 μm gap between sections.

### Immunohistochemistry

Paraffin embedded samples, after deparaffinization and rehydration, were treated by steam heating for antigen retrieval (30 min) using DAKO antigen retrieval solution (DAKO, Carpenteria, CA). Slides were washed using Tris Buffered Saline (TBS) twice. Endogenous peroxidase was inhibited by immersing the slides in a 3% hydrogen peroxide solution for 10 min. Slides were then washed twice in TBS. The sections were incubated with primary antibody against GDNF 1 hour at room temperature. Slides were washed twice with TBS and consecutively incubated with biotinylated secondary antibody for 30 min. Slides were washed twice with TBS and incubated with DAB for 5 min. Slides were washed twice again with distilled water. Immediately after staining, slides were counterstained with hematoxylin for 1 min. Slides were rinsed for 1 second with distilled water and dehydrated for 1–2 seconds each with 90–100% isopropanol. Finally, samples were immersed in xylene for 10 min each and mounted using Permount (Fisher Scientific, Pittsburg, PA).

### Immunofluorescent microscopy

The transversal frozen sections (10 μm) of sciatic nerves were dried and incubated in blocking buffer containing 1.5% normal goat serum and 0.2% Triton X-100 in PBS. The slides were washed twice with PBS, incubated with the primary antibodies (OX-42, phospho-p38, NeuN) at 4°C, 3 days, followed by repeated washing with PBS, and replaced in secondary antibodies conjugated with Alexa 488 or Cy3 for 3 hours at room temperature.

### TUNEL test

The mode of cell death induced by CCI was determined by morphological observations done with terminal deoxynucleotidyl transferase (TdT)-mediated dUTP nick end labelling (TUNEL) assay. Briefly, the tissues were fixed with 4% methanol-free paraformaldehyde at 4 °C and washed with phosphate-buffered saline (PBS) for 30 min. An equilibrium buffer (0.1 ml) was added to each of the slides and covered with parafilm for 10 min at 37 °C. A mixture of 1 μl TdT enzyme, 5 μl nucleotide mix and 45 μl equilibrium buffer was prepared in the dark and 50 μl of the mixture was added onto each slide. Slides were incubated in the dark for 1 or 2 h at 37 °C. SSC (2X) was added for 15 min at room temperature to stop the TdT enzyme reaction. The unbound fluorescent-12-dUTP was removed by washing with PBS. The slides were then immersed in propidium iodide for 15 min in the dark to stain the cells. Slides were dried after rinsing with de-ionized water and cover slips were later overlaid on the cell area of the slides.

### Western blot

For protein extraction, each single hemi-cord segment was homogenized in protein lysis buffer in the presence of protease inhibitors and incubated on ice for 10 min. Samples were centrifuged at 13,000×rpm for 30 min at 4 °C. Total protein content was determined in the supernatants by the Bio-Rad DC Protein Assay Kit. For Western blot analysis, equal amounts of total protein were separated by sodium dodecyl-sulfate polyacrylamide gel electrophoresis (SDS-PAGE; 12%) and transferred onto PVDF membranes. After blocking for 1 hour at room temperature in Tris-buffered saline containing 0.05% Tween 20 (TBST) and 5% non-fat milk, the membranes were incubated overnight at 4°C with the primary antibody including GDNF (1∶100 dilutions; Santa Cruz Biotechnology Inc.); GDNFRa-1(1∶100 dilution; R&D Systems Inc.); ERK, p-ERK, p38, phospho-p38, AIF, caspase-3, cleaved caspase-3, caspase-9, cleaved caspase-9, Beclin-1, MMP-2, MMP-9, iNOS, nNOS, eNOS, PARP, cleaved PARP, PKCγ, PKCδ (1: 1,000 dilution; Cell Signaling Technology); ED1, SPECTRIN and cleaved SPECTRIN (1∶200 dilutions; Santa Cruz Biotechnology Inc.) directed against the protein of interest. After several washes, an appropriate HRP conjugated secondary antibody (1∶5000; Vector Laboratories) was applied for 1 hour at room temperature. Peroxidase activity was visualized using the ECL Western Blotting Detection kit and X-ray films. Quantification of western blots and TUNEL staining were the average band intensities and/or cells with positive staining of chosen antibodies of three independent experiments were determined using ImageJ and plotted.

### Data Analyses

Comparisons within groups were made by using one-way analysis of variance (ANOVA). The comparisons across groups were accomplished with one-way ANOVA and, if significant, discrete comparisons were accomplished using Tukey's method for post-hoc testing. A p value of less than 0.05 was considered statistically significant. Data were expressed as mean ± SEM.

## Results

### GDNF attenuates CCI-induced allodynia and hyperalgesia

**Figure 1:**
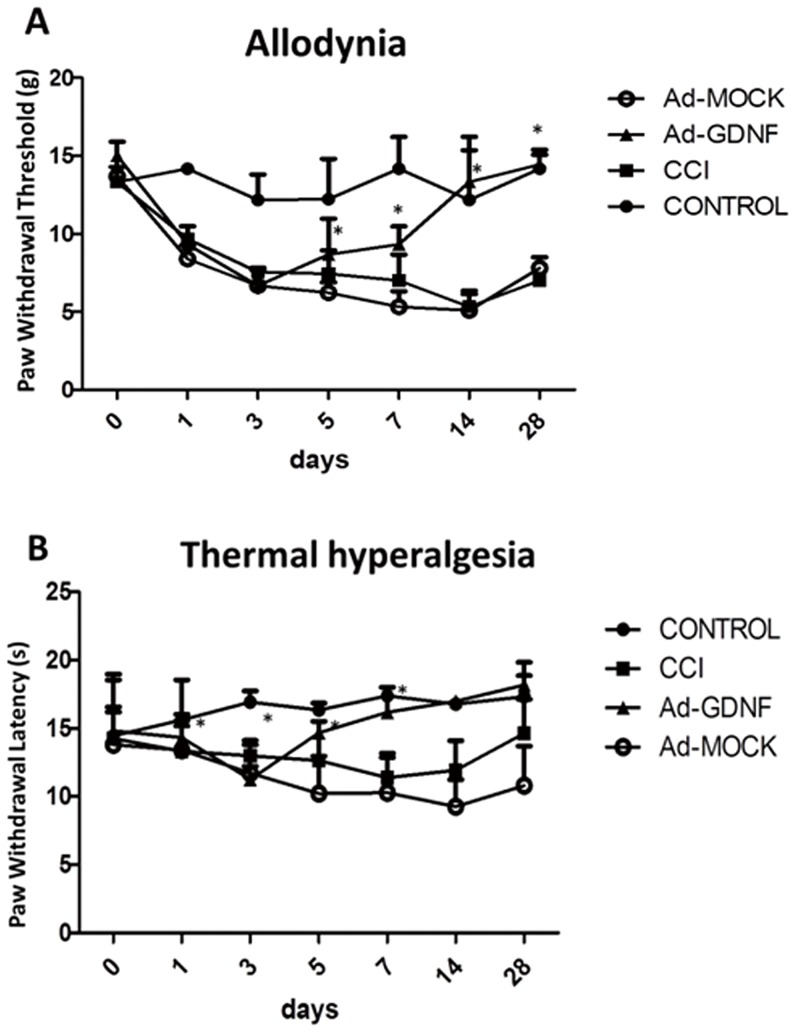
The effect of intramuscular delivery of Ad-GDNF on allodynia (A) and thermal hyperalgesia (B) in the CCI model. *P<0.05 compared with the CCI group at each time point.

To measure the seriousness of neuropathic pain, the Von Frey filament and hot-plantar test were used to establish the animal model. The result showed that CCI induced allodynia and thermal hyperagia at day 1 after surgery (Fig. 1A & 1B). Allodynia was maintained 28 days in CCI group (Fig.1A), whereas thermal hyperalgesia was maintained 14 days but returned to the same level as the control group at day 28 (Fig. 1B). At day 28, rats in the CCI group showed a significantly lower weight for the left hind paw corresponding to the sites where the stimuli were applied (at site of sciatic nerve constriction) compared to controls. Allodynia and thermal hyperalgesia did not differ significantly between the CCI and Ad-MOCK group. Ad-GDNF started to significantly alleviate both allodynia and thermal hyperalgesia associated with CCI at day 5 after surgery, but showed no effect at day 1 and 3 (Fig. 1A & 1B). Therefore, we further investigated the molecular changes underlying the beneficial effect of Ad-GDNF at day 5 in our study.

### GDNF inhibits matrix metalloproteinase expression on SCDH

**Figure 2:**
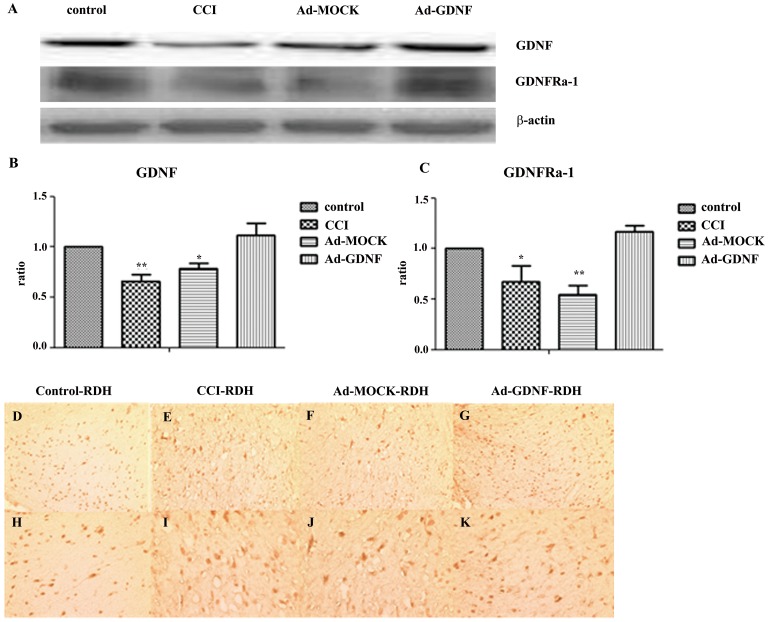
Immunoblotting showing protein expression level with respect to GDNF and its receptor. Western blot analysis showing the expression levels of GDNF and its receptor, GDNFRa-1, under control, CCI, and ipsilateral SCDH with intramuscular injection of Ad-MOCK or Ad-GDNF (A). The expression levels of GDNF and GDNFRa-1 with respect to each tested group were shown as bar charts of relative ratio (B–C). Immunohistochemical (D–K) staining was used to confirm GDNF expression. (D–G: 200X magnification, H–K: 400X magnification) *P<0.05 **P<0.01 compared with control group.

**Figure 3:**
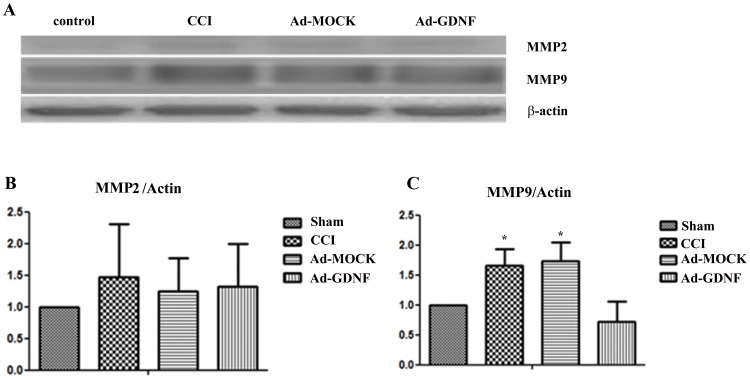
Immunoblotting showing protein expression level with respect to MMP-2 and MMP-9. Western blot analysis showing the expression levels of MMP-2 and MMP-9 in control, CCI, and ipsilateral SCDH with intramuscular injection of Ad-MOCK or Ad-GDNF (A). The expression levels of MMP-2 and MMP-9 with respect to each tested group were shown as bar charts of relative ratio normalized with the expression level of β-actin (B–C). *P<0.05 compared with control group.

To check GDNF and GDNFRa1 protein expression on the SCDH after adenovirus-mediated GDNF was delivered by intramuscular injection, samples prepared from the ipsilateral SCDH at day 5 after surgery were immunoblotted. GDNF and GDNFRa1 expression in both the CCI and Ad-MOCK group was significantly lower than control. After delivery of Ad-GDNF at day 1 after surgery, GDNF and GDNFRa1 expression returned to control levels at day 5 (Fig. 2A–C). Immunohistochemical analysis of GDNF expression was consistent with the results from immunoblotting (Fig. 2D–K). Since Kawasaki Y et al. reported that MMP-9 induces neuropathic pain through interleukin-1β cleavage and microglial activation at early onset [20], we also analyzed MMP-2 and MMP-9 expression. We found no significant inter-group difference between any two groups for MMP-2 expression in the SCDH at day 5. In contrast, in the CCI group the MMP-9 expression was significantly higher than that of controls, but no different than the Ad-MOCK group. Administering Ad-GDNF did inhibit MMP-9 expression, which possibly contributed to the attenuation of neuropathic pain (Fig. 3A–C).

### GDNF decreases activated microglia and expression of inflammatory factors from CCI on SCDH

**Figure 4:**
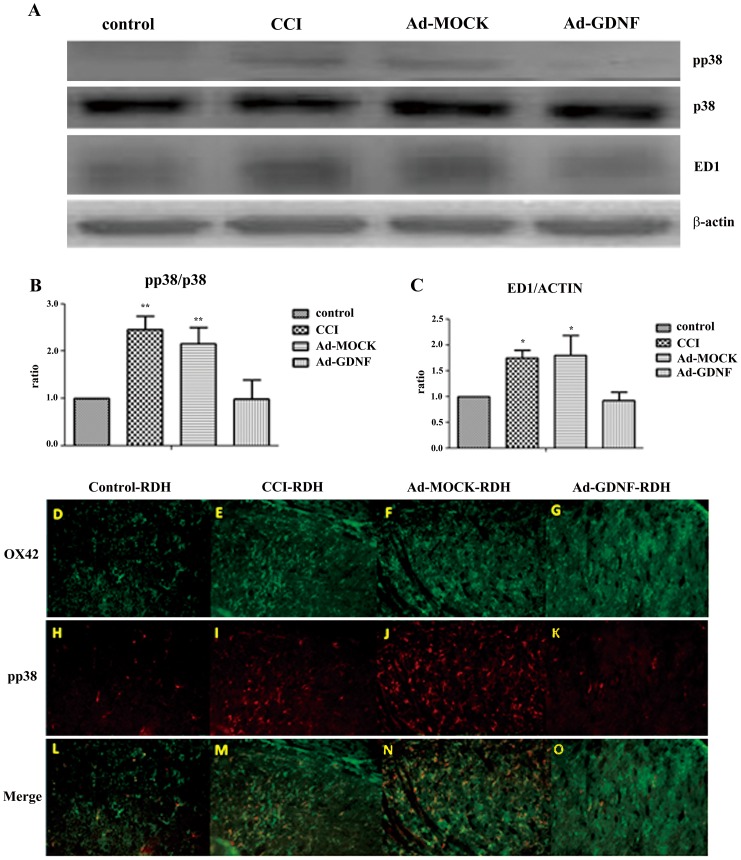
Immunoblotting showing protein expression level with respect to phosphor-p38, p38 and ED-1. Western blot analysis showing the expression levels of phospho-p38, p38 and ED-1 in control, CCI, and ipsilateral SCDH with intramuscular injection of Ad-MOCK or Ad-GDNF (A). The expression levels of phospho-p38 and ED-1 with respect to each tested group were shown as bar charts of relative ratio normalized with expression levels of p38 and β-actin, respectively (B-C). *P<0.05, **P<0.01 compared with the Ad-GDNF group. Double immunofluorescence staining of OX42 (D–G), a microglia marker, and phosphor-p38 (H–K) in different tested groups. The expression levels with respect to OX42 and phospho-p38 were obviously enhanced after CCI, but phospho-p38 was no longer highly expressed after administration of Ad-GDNF as shown in merged images (L–O).

**Figure 5:**
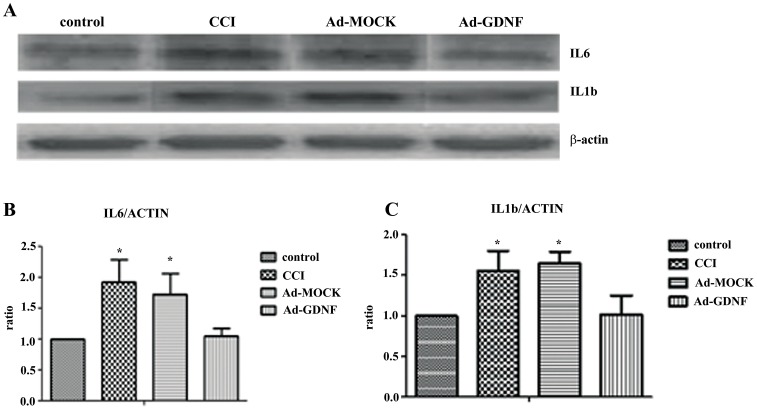
Immunoblotting showing protein expression level with respect to IL-6 and IL-1β. Western blot analysis showing the expression levels of IL-6 and IL-1β in control, CCI, and ipsilateral SCDH with intramuscular injections of Ad-MOCK or Ad-GDNF (A). The expression levels of IL-6 and IL-1β with respect to each tested group were shown as bar charts of relative ratio normalized with the expression levels of β-actin (B–C). *P<0.05, **P<0.01 compared with control group.

A previous study reported that microglia are activated by phosphorylation of p38 and ERK1/2 following peripheral nerve injury including CCI [21]. To confirm this, we detected phosphorylation of p38 and stained for ED-1 expression (microglia marker). Western blot showed that p38 but not ERK1/2 (data not shown) was phosphorylated. In addition, ED-1 expression was elevated in the CCI group. These results may indicate that CCI induced microglia activation and proliferation through the phosphorylation of p38. In contrast, in the Ad-GDNF group the expression of phosphorylated-p38 and ED-1 was significantly lower than in the CCI and Ad-MOCK groups (Fig 4A–C). Double immunofluorescent staining for phosphorylated-p38 and OX42, a microglia marker, showed that expression levels with respect to OX42 and phosphorylated p38 were obviously enhanced after CCI, but phosphorylated p38 was no longer highly expressed after administration of Ad-GDNF (Fig 4D–N). These results revealed CCI-induced phosphorylation of p38 on microglia at SCDH, which returned to normal after GDNF delivery. In CNS, microglia is not only a support cell but is also involved in immune regulation. Microglia activation releases pro-inflammatory cytokines such as TNF-α, IL-1β and IL-6. We examined expression with respect to IL-6 and IL-1β in the different tested groups. Immunoblotting showed that CCI up-regulated IL-6 and IL-1β in the SCDH. After treatment with Ad-GDNF, IL-6 and IL-1β protein expression in the Ad-GDNF group was significantly lower than in the CCI and Ad-MOCK groups, but not the control group (Fig. 5A–C).

### GDNF prevents CCI-induced programmed cell death on SCDH

**Figure 6:**
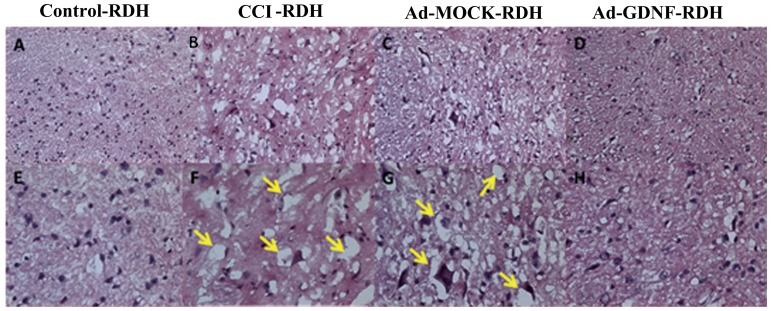
The result of Hematoxylin-Eosin staining (H&E staining) in detecting the morphological changes after administrated with Ad-GDNF. The morphological charges in tight junctions of ipsilateral SCDH among the different tested groups (A–D: 200X, E–G: 400X). Yellow arrows represent possible wallerian degeneration, which was no longer observed after administration of Ad-GDNF.

**Figure 7:**
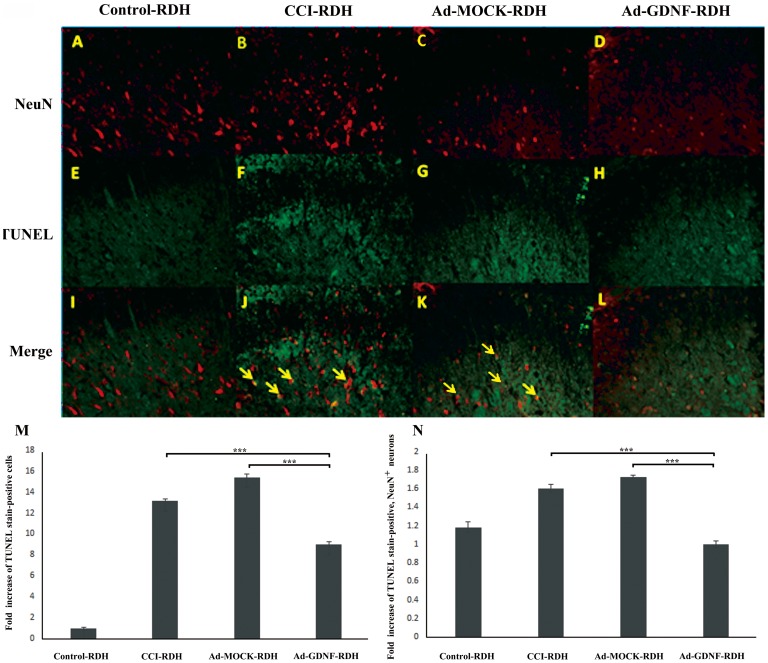
Double immunofluorescent staining of TUNEL and a neuronal cell marker, NeuN, in the rat ipsilateral SCDH in different treatment groups. Tissue samples were detected using antibodies against NeuN (A–D) and TUNEL staining for apoptosis (E–H). The merged images show neuron apoptosis in the ipsilateral SCDH (I–L). Yellow arrows indicate TUNEL-positive neurons. The bar chart with respect to fold increase of TUNEL staining positivity (M) and double labeling (TUNEL and NeuN, N) revealed that apoptotic events triggered by CCI were attenuated by Ad-GDNF.

**Figure 8:**
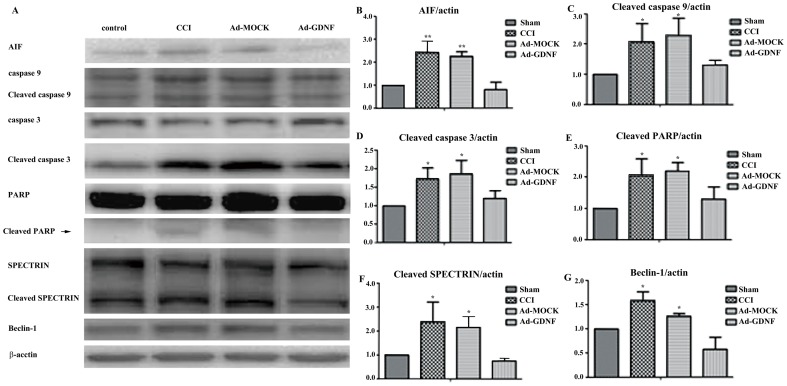
Immunoblotting showing protein expression level with respect to apoptotic and autophagic marker. Western blot analysis of the effect of CCI on the expression of AIF, caspase-9, cleaved caspase-9, caspase-3, cleaved caspase-3, PARP, cleaved PARP, SPECTRIN, cleaved SPECTRIN, and Beclin-1 on ipsilateral SCDH by intramuscular injection with adenovirus plus GDNF gene (A). Ratios of AIF, cleaved caspase-9, cleaved caspase-3, cleaved PARP, cleaved SPECTRIN, Beclin-1 with β-actin on ipsilateral SCDH were measured using western blot analysis (B–G). *P<0.05, **P<0.01 compared with the CCI group.

It had been reported that inflammatory factors induced wallerian degeneration at the lesion site following peripheral nerve injury [22].We also observed SCDH tissue loss by H&E staining after CCI and this hallmark was not recovered by Ad-MOCK administration (Fig. 6F&G). We hypothesized that the tissue loss is possibly due to neuronal reduction caused by programmed cell death. To address this question, we used terminal deoxynucleotidyl transferase dUTP nick end labeling (TUNEL) and immunofluorescent microscopy (Fig.7A–H). We found a significant difference between the CCI, Ad-MOCK and Ad-GDNF group by TUNEL staining (Fig.7 F–H&M), as well as double labeling of TUNEL and NeuN (Fig. 7J–L&N). These results may indicate that SCDH neuron cells underwent apoptosis after CCI and that this phenomenon was reversed by Ad-GDNF. To confirm this finding, we also detected the expressions of several apoptotic proteins. Based on our data, the expressions of apoptosis inducing factor (AIF), cleaved caspase-9, cleaved caspase-3, cleaved Poly (ADP-ribose) polymerase (PARP), cleaved SPECTRIN and Beclin-1 were enhanced in the CCI group (Fig. 8A–G). Interestingly, expression levels of these proteins were attenuated to control group levels after administration of Ad-GDNF. These results suggested that adenoviral-mediated delivery of GDNF successfully inhibited CCI-induced apoptosis in the SCDH (Fig. 8A–G).

### GDNF blocks CCI-induced cellular signaling in SCDH

**Figure 9:**
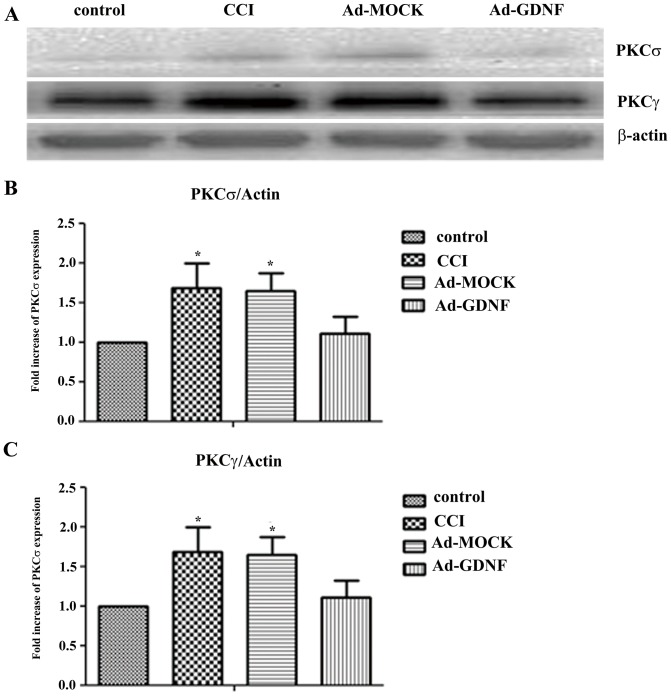
Immunoblotting showing protein expression level with respect to PKCδ and PKCγ. Western blot analysis showing the expression levels of PKCδ and PKCγ in control, CCI, and ipsilateal SCDH with intramuscular injection with Ad-MOCK or Ad-GDNF (A). The expression levels of PKCδ and PKCγ with respect to each tested group were shown as bar charts of relative ratio normalized with the expression levels of β-actin (B–C). *P<0.05, **P<0.01 compared with the Ad-GDNF group.

**Figure 10:**
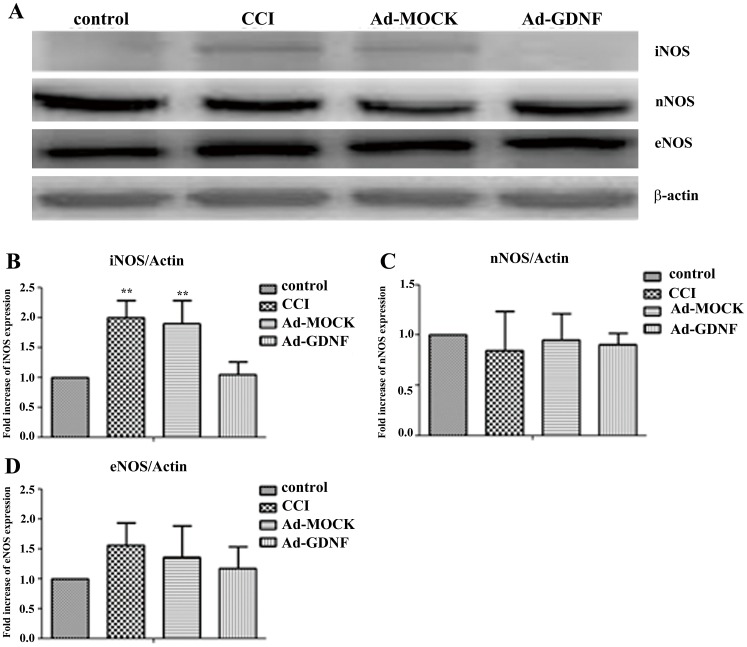
Immunoblotting showing protein expression level with respect to different NOS isoform. Western blot analysis showing the expression levels of iNOS, nNOS and eNOS in control, CCI, and ipsilateral SCDH with intramuscular injection with Ad-MOCK or Ad-GDNF (A). The expression levels of iNOS, nNOS and eNOS with respect to each tested group were shown as bar charts of relative ratio normalized with the expression levels of β-actin (B–D). **P<0.01 compared with Ad-GDNF group.

Mao J et al. reported that PKCγ was increased after CCI [23]. In our results, consistent with previous reports, CCI increased both PKCδ and PKCγ protein expression, but in the Ad-GDNF group expression with respect to PKCδ and PKCγ was significant lower than the Ad-MOCK and CCI group, respectively (Fig. 9A–C). These data clearly indicated that GDNF modulated both PKCδ and PKCγ protein expression on the SCDH after CCI. In addition to examining PKC signaling, we also detected the expressions of NOS family proteins including iNOS, nNOS, and eNOS, since NMDA/PKC signaling was associated with NOS expression. Among these three NOS, only iNOS was increased following CCI and this effect was reversed by Ad-GDNF (Fig. 10B). These data suggested that GDNF may have a role in attenuating CCI-induced PKC/iNOS signaling associated with its neuroprotective effect in the SCDH.

## Discussion

In the CCI-induced nerve injury animal model, microglia activation and abnormal pro-inflammatory cytokine profiling are suggested to be crucial in maintaining neuropathic pain. In fact, microglia are derived from myeloid precursor cells in the periphery and penetrate the CNS during embryogenesis. Microglia are the resident macrophages in the CNS, and mediate signaling crosstalk between peripheral and CNS nerves. Moreover, microglia are also important in CNS neuroinflammation. Mechanical or biochemical stressor insults affecting CNS homeostasis usually induce rapid responses in microglia morphology, gene expression profile and functional behavior and these events are collectively termed ‘microgliosis’. Interestingly, damage to the nervous system outside the CNS, such as axotomy of a peripheral nerve, can lead to microgliosis in the spinal cord. In addition, it is also reported that peripheral nerve injury nociceptive inputs from sensory neurons appear to be critical for triggering the development of spinal microgliosis. CCI-induced neuropathic pain is also associated with microgliosis in the SCDH [24]. However, the physiological role of microglia in spinal cord circuitry development and pain transmission remains to be investigated.

Increasing evidence suggests the important role of spinal cord microglia in the genesis of persistent pain, by releasing the proinflammatory cytokines tumor necrosis factor-alpha (TNFα), Interleukine-1beta (IL-1β), and brain derived neurotrophic factor (BDNF). Nerve injury-induced microglial activation occurs by phosphorylation of MAP kinases such as p38 MAPK kinase and extracellular signal-related kinase (ERK) isoforms 1 and 2, and Src-family kinases [21], [25]–[27]. The morphological changes associated with microgliosis may also be mediated by the activation of ERK/MAPK [28]. A number of signaling pathways such as neuregulin-1, matrix metalloproteases (e.g. MMP-9) and multiple chemokines enable direct communication between injured primary afferents and microglia. According to current knowledge regarding p38 MAPK signaling and increased the pain sensitivity, several molecules have been reported to activate p38, such as TNFα [29], IL-1β[30], CCL2[31], MCP-1, CX3CL1[32], iNOS[33], MMP-9[20], P2X4 and P2X7. Some of these microglial activators, such as ATP, CCL2, fractalkine, and MMP-9, are suggested to be released from primary afferent neurons [34], [35]. When microglia are activated, the p38 pathway induces expression of molecules such as NFκB, COX2, iNOS, BDNF, TNFα, IL-1β, and IL-6 [30], [36], [37]. p38 activation in microglia also results in increased release of BDNF and TNFα [38]. Microglial production of proinflammatory cytokines can further recruit additional microglia, activate surrounding astrocytes, and promote the sensitization of central nervous system nociceptive circuits.

We observed CCI induced activation of microglia by phosphorylation of p38 rather than ERK. Spinal microglial activation in both dorsal and ventral horns peaked 1 week after injury and returned after several weeks. Our results support the idea that that microglia affect inflammatory reactions at an early stage. Yasuhiko Kawasaki et al. reported that after spinal nerve ligation (SNL), MMP-9 induced neuropathic pain through interleukin-1 cleavage and microglial activation at early times, whereas MMP-2 maintained neuropathic pain through interleukin-1 cleavage and astrocyte activation at later times [20]. In our study, MMP-9 was induced at day 5 after CCI, and MMP-2 showed no effect. Microglial activation might be just the first step in a cascade of immune responses in the CNS. Zhuang et al. showed the activation of ERK in neurons, then microglia, and then astrocytes in a neuropathic pain model [27]. Microglia may initiate neuropathic pain, and astrocytes probably respond to maintain neuropathic pain. Microglial MAP kinases can be activated by IL-1β and TNF-α, inducing, via transcription factors such as NFκB, additional production of IL-1β, TNF-α, IL-6, IL-10, TGF-β, PGE2, BDNF, and cathepsin S and promoting the deleterious effects of microglial infiltration and phagocytosis in neuropathic pain. Blocking the signaling pathways mediated by IL-1β or IL-6 diminishes behaviors related to neuropathic pain [39]. In this study, GDNF inhibited the activation of microglia and IL-1βand IL-6 release. This can be one of reasons for the decline in pain behaviors after Ad-GDNF administration.

The present study explored how CCI-induced peripheral nerve injury can cause type I (apoptosis) and II (autophagy) programmed cell death. These molecular events can also be attenuated by adenoviral-mediated GDNF delivery. Consistent with other studies, the present data revealed that pro-inflammatory cytokine signaling, such as TNFα, p38, and MMP-9, may contribute to apoptosis induction in the SCDH after CCI. Previous studies suggested that either apoptosis or inflammation plays an important role in neuropathic pain. Peripheral nerve injury produces neuron apoptosis in the dorsal horn of the spinal cord [40]–[42] and DRG [43], [44]. Specifically, apoptosis in satellite glial cells (SGC) (not in neurons) of CCI rat ipsilateral dorsal root ganglia (DRG) at day 30 after injury was revealed [45].

Cleaved caspase-3 and caspase-9 expression is the effector with respect to apoptosis and post-mitochondrial apoptosis. Our results showed that GDNF can reduce CCI-induced caspase expression and activation, which may be associated with relief of pain behavior. This data suggests that caspase signaling pathways are involved in pain development. Apoptosis inducing factor (AIF) is used as a marker of mitochondrial apoptosis. Both caspases and AIF can trigger SPECTRIN cleavage by activating cleaved Poly (ADP-ribose) polymerase (PARP). All of them are apoptosis markers. In our results, CCI induced the expression and activation of chosen markers involved in either mitochondrial or non-mitochondrial apoptosis. Moreover, using adenovirally delivered GDNF successfully attenuated apoptotic protein expression.

Autophagy is an intracellular membrane trafficking pathway controlling the delivery of cytoplasmic material to the lysosomes for degradation. It plays an important role in cell homeostasis in both normal settings and abnormal or stressful conditions[46]. LC3B and Beclin-1 are autophagy markers involved in different stages of autophagy. Previous studies reported that L5 spinal nerve ligation induced autophagy in the SCDH. Both LC3 and Beclin-1 are observed to be significantly elevated in the ipsilateral L5 spinal dorsal horn on day 14 following spinal nerve ligation. These two proteins are mainly located at GABAergic interneurons of the spinal dorsal horn after SNL, indicating that autophagic disruption in GABAergic interneurons and astrocytes following peripheral nerve injury might be involved in the induction and maintenance of neuropathic pain [47]. Besides, the mTOR pathway (autophagy-associated) is also reported to be activated in the SCDH in CCI-induced neuropathic pain, and the intrathecal injection of rapamycin can reduce mechanical allodynia [48]. In this study, CCI-induced peripheral nerve injury did lead to autophagy induction on the SCDH and GDNF prevented the elevated expression of Beclin-1 due to CCI-induced nerve injury.

In conclusion, intramuscular injection with Ad-GDNF not only attenuates neuropathic pain but also protects cells from neuropathic pain-associated programmed cell death (microglia inactivation through down-regulating IL-6, IL-1β, p38 and MMP-9). In addition, administration of GDNF also enhanced expression of inducible nitric oxide synthases by modulating the PKC pathway in the SCDH following chronic constriction injury. Adenoviral GDNF-based gene therapy may be an alternative therapeutic approach for treating neuropathic pain in patients.

Although our results provide evidence that GDNF can be applied to attenuate CCI-induced neuropathic pain, the experimental limitations should be noted. First, CCI-induced nerve injury was recently associated with autophagy induction. Many proteins are used as hallmarks of autophagy, such as ATG family proteins, p62, Beclin 1 and LC3B. In this study, we only measured Beclin 1 and LC3B to compare the results with the SNL model. Second, for evaluating apoptotic events after CCI-induced nerve injury on the SCDH, we only used TUNEL staining, NeuN staining and immunostaining of apoptotic proteins. This may not fully characterize the apoptosis. Information regarding DNA content and cell-cycle distribution consequent to CCI and Ad-GDNF treatment was not obtained. Third, the lack of another neurotrophic factor such as BDNF, NGF and NT3 as control to compare with results of GDNF limited our conclusions about the therapeutic value of Ad-GDNF. Finally, to discriminate the signaling pathways associated with the beneficial effects attributed to GDNF in attenuating CCI-induced nerve injury, we only directly observed the modulating role of GDNF on target protein expressions rather than introducing any pathway inhibitors.

Our major finding was that adenovirally mediated delivery of GDNF successfully decreased neuropathic pain behaviors and their associated protein expressions. GDNF appears to inhibit microglia activation, pro-inflammatory cytokine production, and at least two types of programmed cell death (apoptosis and autophagy). Future work on signaling pathways and cross-talk consequent to GDNF administration will provide further insights into its therapeutic action in terms of CCI-induced neuropathic pain attenuation, and provide a starting point for developing new strategies for pain control.

## References

[1] Lekan HA, Carlton SM, Coggeshall RE (1996) Sprouting of A beta fibers into lamina II of the rat dorsal horn in peripheral neuropathy. Neurosci Lett 208: 147–150. 8733291 10.1016/0304-3940(96)12566-0

[2] Woolf CJ, Shortland P, Coggeshall RE (1992) Peripheral nerve injury triggers central sprouting of myelinated afferents. Nature 355: 75–78. 1370574 10.1038/355075a0

[3] Shortland P, Wang HF, Molander C (1999) Distribution of transganglionically labelled soybean agglutinin primary afferent fibres after nerve injury. Brain Res 815: 206–212. 9878739 10.1016/s0006-8993(98)01152-4

[4] Bridges D, Thompson SW, Rice AS (2001) Mechanisms of neuropathic pain. Br J Anaesth 87: 12–26. 11460801 10.1093/bja/87.1.12

[5] Airaksinen MS, Saarma M (2002) The GDNF family: signalling, biological functions and therapeutic value. Nat Rev Neurosci 3: 383–394. 11988777 10.1038/nrn812

[6] Bennett DL, Michael GJ, Ramachandran N, Munson JB, Averill S, et al (1998) A distinct subgroup of small DRG cells express GDNF receptor components and GDNF is protective for these neurons after nerve injury. J Neurosci 18: 3059–3072. 9526023 10.1523/JNEUROSCI.18-08-03059.1998 PMC6792585

[7] Boucher TJ, Okuse K, Bennett DL, Munson JB, Wood JN, et al (2000) Potent analgesic effects of GDNF in neuropathic pain states. Science 290: 124–127. 11021795 10.1126/science.290.5489.124

[8] Hao S, Mata M, Wolfe D, Huang S, Glorioso JC, et al (2003) HSV-mediated gene transfer of the glial cell-derived neurotrophic factor provides an antiallodynic effect on neuropathic pain. Mol Ther 8: 367–375. 12946309 10.1016/s1525-0016(03)00185-0

[9] Ramer MS, Priestley JV, McMahon SB (2000) Functional regeneration of sensory axons into the adult spinal cord. Nature 403: 312–316. 10659850 10.1038/35002084

[10] Eslamboli A (2005) Assessment of GDNF in primate models of Parkinson's disease: comparison with human studies. Rev Neurosci 16: 303–310. 16519007 10.1515/revneuro.2005.16.4.303

[11] Gill SS, Patel NK, Hotton GR, O'Sullivan K, McCarter R, et al (2003) Direct brain infusion of glial cell line-derived neurotrophic factor in Parkinson disease. Nat Med 9: 589–595. 12669033 10.1038/nm850

[12] Manabe Y, Nagano I, Gazi MS, Murakami T, Shiote M, et al (2002) Adenovirus-mediated gene transfer of glial cell line-derived neurotrophic factor prevents motor neuron loss of transgenic model mice for amyotrophic lateral sclerosis. Apoptosis 7: 329–334. 12101392 10.1023/a:1016123413038

[13] Wang LJ, Lu YY, Muramatsu S, Ikeguchi K, Fujimoto K, et al (2002) Neuroprotective effects of glial cell line-derived neurotrophic factor mediated by an adeno-associated virus vector in a transgenic animal model of amyotrophic lateral sclerosis. J Neurosci 22: 6920–6928. 12177190 10.1523/JNEUROSCI.22-16-06920.2002 PMC6757879

[14] Bennett GJ, Xie YK (1988) A peripheral mononeuropathy in rat that produces disorders of pain sensation like those seen in man. Pain 33: 87–107. 2837713 10.1016/0304-3959(88)90209-6

[15] Tai MH, Cheng H, Wu JP, Liu YL, Lin PR, et al (2003) Gene transfer of glial cell line-derived neurotrophic factor promotes functional recovery following spinal cord contusion. Exp Neurol 183: 508–515. 14552891 10.1016/s0014-4886(03)00130-4

[16] Giraud C, Winocour E, Berns KI (1995) Recombinant junctions formed by site-specific integration of adeno-associated virus into an episome. J Virol 69: 6917–6924. 7474109 10.1128/jvi.69.11.6917-6924.1995 PMC189609

[17] Hargreaves K, Dubner R, Brown F, Flores C, Joris J (1988) A new and sensitive method for measuring thermal nociception in cutaneous hyperalgesia. Pain 32: 77–88. 3340425 10.1016/0304-3959(88)90026-7

[18] Eftekhari S, Edvinsson L (2011) Calcitonin gene-related peptide (CGRP) and its receptor components in human and rat spinal trigeminal nucleus and spinal cord at C1-level. BMC Neurosci 12: 112. 22074408 10.1186/1471-2202-12-112 PMC3282678

[19] Yamazaki K, Wakasugi N, Tomita T, Kikuchi T, Mukoyama M, et al (1988) Gracile axonal dystrophy (GAD), a new neurological mutant in the mouse. Proc Soc Exp Biol Med 187: 209–215. 3340629 10.3181/00379727-187-42656

[20] Kawasaki Y, Xu ZZ, Wang X, Park JY, Zhuang ZY, et al (2008) Distinct roles of matrix metalloproteases in the early- and late-phase development of neuropathic pain. Nat Med 14: 331–336. 18264108 10.1038/nm1723 PMC2279180

[21] Jin SX, Zhuang ZY, Woolf CJ, Ji RR (2003) p38 mitogen-activated protein kinase is activated after a spinal nerve ligation in spinal cord microglia and dorsal root ganglion neurons and contributes to the generation of neuropathic pain. J Neurosci 23: 4017–4022. 12764087 10.1523/JNEUROSCI.23-10-04017.2003 PMC6741086

[22] George A, Buehl A, Sommer C (2004) Wallerian degeneration after crush injury of rat sciatic nerve increases endo- and epineurial tumor necrosis factor-alpha protein. Neurosci Lett 372: 215–219. 15542243 10.1016/j.neulet.2004.09.075

[23] Mao J, Price DD, Phillips LL, Lu J, Mayer DJ (1995) Increases in protein kinase C gamma immunoreactivity in the spinal cord dorsal horn of rats with painful mononeuropathy. Neurosci Lett 198: 75–78. 8592645 10.1016/0304-3940(95)11975-3

[24] Tsuda M, Inoue K, Salter MW (2005) Neuropathic pain and spinal microglia: a big problem from molecules in “small” glia. Trends Neurosci 28: 101–107. 15667933 10.1016/j.tins.2004.12.002

[25] Katsura H, Obata K, Mizushima T, Sakurai J, Kobayashi K, et al (2006) Activation of Src-family kinases in spinal microglia contributes to mechanical hypersensitivity after nerve injury. J Neurosci 26: 8680–8690. 16928856 10.1523/JNEUROSCI.1771-06.2006 PMC6674378

[26] Svensson CI, Fitzsimmons B, Azizi S, Powell HC, Hua XY, et al (2005) Spinal p38beta isoform mediates tissue injury-induced hyperalgesia and spinal sensitization. J Neurochem 92: 1508–1520. 15748168 10.1111/j.1471-4159.2004.02996.x

[27] Zhuang ZY, Gerner P, Woolf CJ, Ji RR (2005) ERK is sequentially activated in neurons, microglia, and astrocytes by spinal nerve ligation and contributes to mechanical allodynia in this neuropathic pain model. Pain 114: 149–159. 15733640 10.1016/j.pain.2004.12.022

[28] Calvo M, Zhu N, Grist J, Ma Z, Loeb JA, et al.. (2011) Following nerve injury neuregulin-1 drives microglial proliferation and neuropathic pain via the MEK/ERK pathway. Glia. 10.1002/glia.21124 PMC3222694 21319222

[29] Svensson CI, Schafers M, Jones TL, Powell H, Sorkin LS (2005) Spinal blockade of TNF blocks spinal nerve ligation-induced increases in spinal P-p38. Neurosci Lett 379: 209–213. 15843065 10.1016/j.neulet.2004.12.064

[30] Sung CS, Wen ZH, Chang WK, Chan KH, Ho ST, et al (2005) Inhibition of p38 mitogen-activated protein kinase attenuates interleukin-1beta-induced thermal hyperalgesia and inducible nitric oxide synthase expression in the spinal cord. J Neurochem 94: 742–752. 16033422 10.1111/j.1471-4159.2005.03226.x

[31] Abbadie C, Lindia JA, Cumiskey AM, Peterson LB, Mudgett JS, et al (2003) Impaired neuropathic pain responses in mice lacking the chemokine receptor CCR2. Proc Natl Acad Sci U S A 100: 7947–7952. 12808141 10.1073/pnas.1331358100 PMC164693

[32] Zhuang ZY, Kawasaki Y, Tan PH, Wen YR, Huang J, et al (2007) Role of the CX3CR1/p38 MAPK pathway in spinal microglia for the development of neuropathic pain following nerve injury-induced cleavage of fractalkine. Brain Behav Immun 21: 642–651. 17174525 10.1016/j.bbi.2006.11.003 PMC2084372

[33] Tang Q, Svensson CI, Fitzsimmons B, Webb M, Yaksh TL, et al (2007) Inhibition of spinal constitutive NOS-2 by 1400W attenuates tissue injury and inflammation-induced hyperalgesia and spinal p38 activation. Eur J Neurosci 25: 2964–2972. 17561811 10.1111/j.1460-9568.2007.05576.x

[34] Clark AK, Malcangio M (2012) Microglial signalling mechanisms: Cathepsin S and Fractalkine. Exp Neurol 234: 283–292. 21946268 10.1016/j.expneurol.2011.09.012

[35] Ji RR, Xu ZZ, Wang X, Lo EH (2009) Matrix metalloprotease regulation of neuropathic pain. Trends Pharmacol Sci 30: 336–340. 19523695 10.1016/j.tips.2009.04.002 PMC2706286

[36] Svensson CI, Marsala M, Westerlund A, Calcutt NA, Campana WM, et al (2003) Activation of p38 mitogen-activated protein kinase in spinal microglia is a critical link in inflammation-induced spinal pain processing. J Neurochem 86: 1534–1544. 12950462 10.1046/j.1471-4159.2003.01969.x

[37] Ji RR, Suter MR (2007) p38 MAPK, microglial signaling, and neuropathic pain. Mol Pain 3: 33. 17974036 10.1186/1744-8069-3-33 PMC2186318

[38] Wen YR, Tan PH, Cheng JK, Liu YC, Ji RR (2011) Microglia: a promising target for treating neuropathic and postoperative pain, and morphine tolerance. J Formos Med Assoc 110: 487–494. 21783017 10.1016/S0929-6646(11)60074-0 PMC3169792

[39] Wolf G, Gabay E, Tal M, Yirmiya R, Shavit Y (2006) Genetic impairment of interleukin-1 signaling attenuates neuropathic pain, autotomy, and spontaneous ectopic neuronal activity, following nerve injury in mice. Pain 120: 315–324. 16426759 10.1016/j.pain.2005.11.011

[40] Maione S, Siniscalco D, Galderisi U, de Novellis V, Uliano R, et al (2002) Apoptotic genes expression in the lumbar dorsal horn in a model neuropathic pain in rat. Neuroreport 13: 101–106. 11924868 10.1097/00001756-200201210-00024

[41] Moore KA, Kohno T, Karchewski LA, Scholz J, Baba H, et al (2002) Partial peripheral nerve injury promotes a selective loss of GABAergic inhibition in the superficial dorsal horn of the spinal cord. J Neurosci 22: 6724–6731. 12151551 10.1523/JNEUROSCI.22-15-06724.2002 PMC6758148

[42] Whiteside GT, Munglani R (2001) Cell death in the superficial dorsal horn in a model of neuropathic pain. J Neurosci Res 64: 168–173. 11288144 10.1002/jnr.1062

[43] Campana WM, Myers RR (2003) Exogenous erythropoietin protects against dorsal root ganglion apoptosis and pain following peripheral nerve injury. Eur J Neurosci 18: 1497–1506. 14511329 10.1046/j.1460-9568.2003.02875.x

[44] Sekiguchi M, Kobayashi H, Sekiguchi Y, Konno S, Kikuchi S (2008) Sympathectomy reduces mechanical allodynia, tumor necrosis factor-alpha expression, and dorsal root ganglion apoptosis following nerve root crush injury. Spine (Phila Pa 1976) 33: 1163–1169. 18469688 10.1097/BRS.0b013e31817144fc

[45] Schaeffer V, Meyer L, Patte-Mensah C, Eckert A, Mensah-Nyagan AG (2010) Sciatic nerve injury induces apoptosis of dorsal root ganglion satellite glial cells and selectively modifies neurosteroidogenesis in sensory neurons. Glia 58: 169–180. 19565659 10.1002/glia.20910

[46] Mizushima N, Levine B, Cuervo AM, Klionsky DJ (2008) Autophagy fights disease through cellular self-digestion. Nature 451: 1069–1075. 18305538 10.1038/nature06639 PMC2670399

[47] Zhang E, Yi MH, Ko Y, Kim HW, Seo JH, et al (2013) Expression of LC3 and Beclin 1 in the spinal dorsal horn following spinal nerve ligation-induced neuropathic pain. Brain Res 1519: 31–39. 23665054 10.1016/j.brainres.2013.04.055

[48] Zhang W, Sun XF, Bo JH, Zhang J, Liu XJ, et al (2013) Activation of mTOR in the spinal cord is required for pain hypersensitivity induced by chronic constriction injury in mice. Pharmacol Biochem Behav 111C: 64–70. 10.1016/j.pbb.2013.07.017 23948070

